# An *In Vivo* Study on the Feasibility of Catheter-Assisted Pulsed Focused Ultrasound Ablation of Atherosclerotic Plaques

**DOI:** 10.1016/j.ultrasmedbio.2025.06.021

**Published:** 2025-07-18

**Authors:** Abhirup Samaddar, Prakash Adhikari, Dalin Wang, Sa Wu, Jinxi Wang, Marcus Laird Forrest, Xinmai Yang

**Affiliations:** a Institute for Bioengineering Research and Department of Mechanical Engineering, the University of Kansas, Lawrence, KS, USA; b Department of Orthopedic Surgery, the University of Kansas Medical Center, Kansas City, KS, USA; c Department of Pharmaceutical Chemistry, the University of Kansas, Lawrence, KS, USA

**Keywords:** Atherosclerosis, Focused ultrasound, Cavitation, Catheter

## Abstract

**Objective:**

Atherosclerosis is a condition where arteries become constricted due to plaque deposition, impairing blood supply and potentially leading to stroke and other cardiovascular diseases. This study aims to develop a minimally invasive technology that combines focused ultrasound (FUS) with a catheter to remove arterial plaques through the cavitation effect.

**Methods:**

In this investigation, via an *ex vivo* study in atherosclerotic plaque samples, we demonstrated that the onset of cavitation events can be enhanced by placing a catheter in the focal spot of the FUS field. Then, in a balloon injury-induced rabbit atherosclerosis model, FUS treatments with and without a catheter in the FUS field were performed on the femoral artery at 500 kHz with a 4 MPa peak negative pressure (PNP), 10% duty cycle and 100 Hz pulse repetition rate.

**Results:**

In our *ex vivo* study using a catheter with a 0.67 mm diameter in the focal zone of an FUS transducer of 500 kHz center frequency, cavitation was initiated at a PNP of 1.9 MPa, whereas FUS alone required a PNP above 4 MPa to induce cavitation. In our *in vivo* study, FUS-only group demonstrated negligible lumen re-canalization effect by applying 4 MPa PNP, whereas using catheter-assisted FUS therapy resulted in maximum lumen re-canalization using the same PNP. Results were statistically significant between groups (*p* < 0.001).

**Conclusion:**

The results showed that catheter-assisted pulsed FUS therapy was effective in re-canalizing a blocked artery.

## Introduction

Cardiovascular disease (CVD) is responsible for 17 million deaths globally per annum [1]. Atherosclerosis is a common type of CVD, which is characterized by intimal lesions called atheromatous or atherosclerotic plaques that protrude into vessel lumens. Although the pathogenesis of atherosclerosis is not fully understood, a variety of insults including endothelial cell injury, lipid accumulation and smooth muscle cell proliferation are proposed to be involved in the atherosclerotic pathology. Disruption of the fibrous cap of atherosclerotic plaques can lead to thrombosis and vascular occlusion causing clinical problems, such as ischemic stroke and myocardial infarction [2,3]. Several therapeutic procedures exist for reducing the incidence of atherosclerotic CVD. Cholesterol-lowering medications are the most commonly used. Hypolipidemic drugs such as statins can slow down the development of atherosclerosis in the arteries by reducing low-density lipoprotein cholesterol [4]. However, these medications cannot re-canalize a blocked artery. In case that medications are not effective in treating atherosclerosis-related arterial blockages, interventional procedures will be performed. The common interventional procedure for coronary atherosclerosis is percutaneous coronary intervention, also known as angioplasty and stenting [5]. For carotid artery, the atherosclerosis is more often removed surgically by endarterectomy [6], which is invasive but more effective than carotid angioplasty and stenting [7]. For severe atherosclerosis in coronary arteries, also known as multi-vessel disease, a coronary artery bypass grafting can be used to create a bypass around the blockage [8]. In addition, minimally invasive procedures such as orbital atherectomy and excimer laser angioplasty [9] have also been routinely used in clinics for peripheral artery diseases.

Focused ultrasound (FUS) has been used therapeutically for several decades. For example, high-intensity FU (HIFU) is used as a non-invasive approach to induce thermal lesions to treat tumors in the prostate [10], liver and kidney [11,12]; and histotripsy, using a cavitation mechanism, has been used for disintegrating tumors and for blood vessel re-canalization [13,14]. The direct use of FUS is more limited in cardiovascular applications as high-intensity acoustic energy or pressure may induce damage in nearby healthy tissues, including vessel walls [15]. To improve the safety and efficacy of ultrasound-based thrombolysis, ultrasound contrast agents (micro-bubbles) are often injected [16–19].

Although numerous studies have been carried out on using FUS to remove blood clots [17,20–24], only a few investigations have used FUS to deplete atherosclerotic plaques. An *in vivo* study applied HIFU to thermally ablate atherosclerotic plaques non-invasively in swine models [25,26]. However, this approach does not remove the plaque and re-canalize a blocked artery. Another *in vivo* study was conducted in a rabbit atherosclerotic plaque model, using a 5.3 MHz therapeutic transducer along with a bolus of micro-bubbles to enhance cavitation for the plaque ablation process [27]. However, the approach is limited by the amount of micro-bubbles that can be injected and their residence time in the treatment area; in addition, micro-bubbles may migrate toward the proximal or distal arterial wall which increases the risk of arterial lumen damage during FUS exposure [28,29]. Echogenic liposomes, which can encapsulate bioactive gases and therapeutic agents with a potential to be imaged and activated by ultrasound, are recently developed to deliver enhanced penetration and treatment efficacy for atherosclerosis [30]. Again, this strategy requires the injection of exogenous contrast agents. Our recent *ex vivo* study showed that although atherosclerotic plaque could be removed by combining relatively low-pressure FUS (5.45 MPa peak negative pressure (PNP)) with laser energy to induce cavitation, using only FUS, it was difficult to remove lipid materials from an atherosclerotic plaque at low PNP [31].

We recently demonstrated *in vitro* that a catheter can be placed in the FUS field to achieve enhanced cavitation at relatively low PNP [32]. We demonstrated that the placement of a catheter within a 4 MPa PNP FUS field was able to disrupt lipid contents in both artificial and excised patient atherosclerotic plaques. The relatively low acoustic pressure used in this study would be expected to cause minimum to no collateral damage during the application of FUS for atherosclerotic plaque removal. In the current study, the enhancement of FUS using a catheter was examined *in vivo* using a rabbit atherosclerotic plaque model. Atherosclerotic plaques were created in the femoral arteries of rabbits and then treated with FUS at 4 MPa PNP and 10% duty cycle, with or without the assistance of a catheter. Our results demonstrated the feasibility of catheter-assisted pulsed FUS ablation of atherosclerotic plaques *in vivo*.

## Materials and methods

### Experimental setup

**Figure 1.**
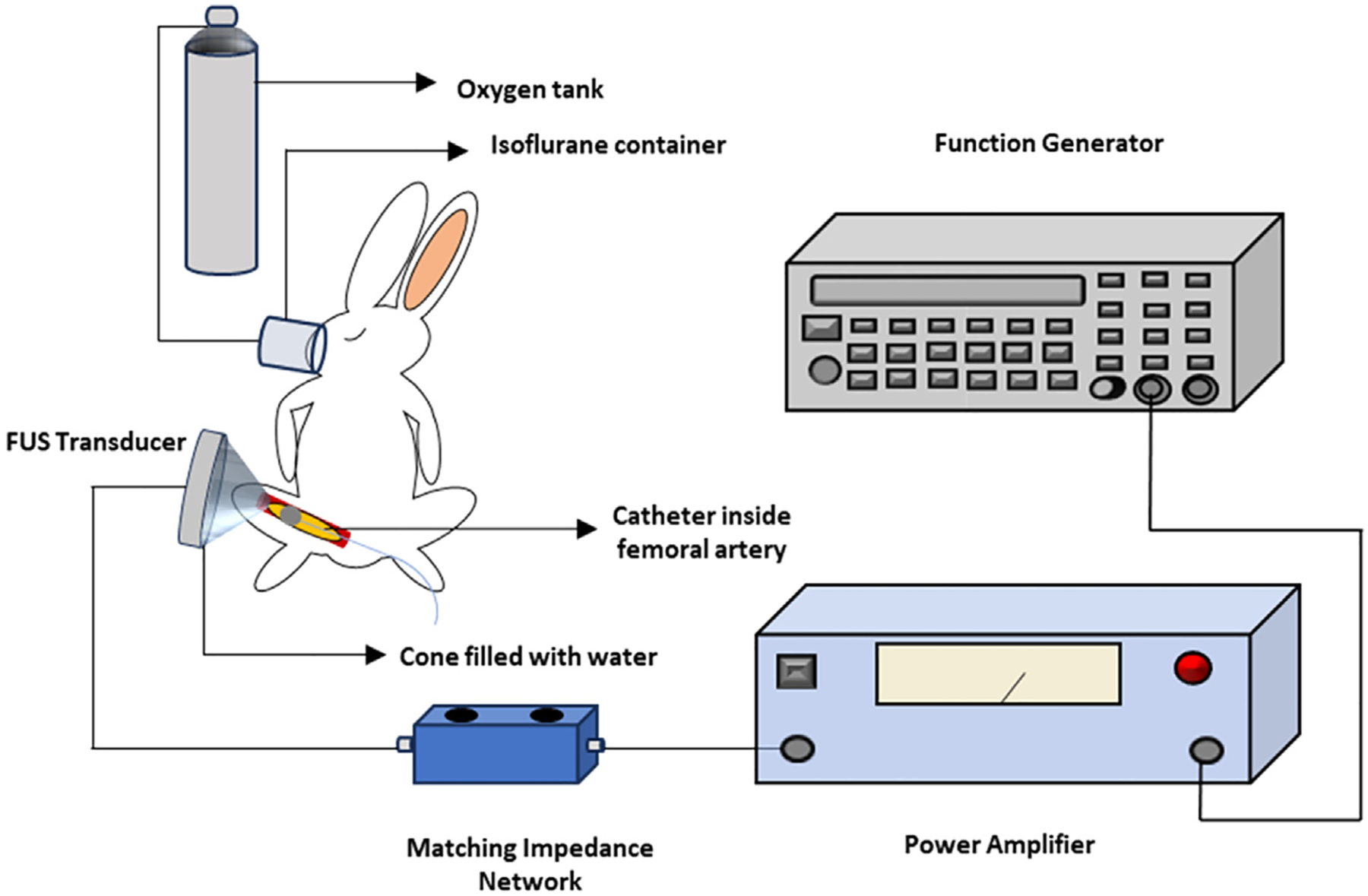
Experimental setup schematic.

The experiment schematic for the catheter-assisted FUS study is shown in Figure 1. In this setup, a therapeutic FUS transducer of 500 kHz central frequency (H-107, Sonic Concepts, Bothell, WA, USA) was used to transmit ultrasound waves to the target. The original 500 kHz signal was produced by a function generator (33250A, Agilent Technologies, Santa Clara, CA, USA), which produced acoustic bursts with 500 sinusoidal cycles at a pulse repetition frequency of 100 Hz. The output of the function generator was passed into a power amplifier (2100 L, Electronics & Innovation Ltd., Rochester, NY, USA), and then the output of the power amplifier was supplied to the FUS transducer through a matching impedance network (Impedance matching network, Sonic Concepts).

A custom-made conical shape cone was attached to the front surface of the FUS transducer to help focus the ultrasound waves and hold degassed and deionized water for ultrasound transmission. Finally, a plastic membrane covered the distal end of the cone to prevent water leakage while allowing ultrasound waves to transmit effectively. The focal zone of the FUS was at the tip of the conical cone. During the treatment process, a catheter was placed in the FUS transducer focal region, which was also the target area. In the *in vivo* study, the catheter was inserted into the left femoral artery of the animal, where atherosclerotic plaques were created and targeted by the FUS transducer. The FUS transducer has a focal length of 21.42 mm and a focal width of 3.02 mm, which would cover the entire diameter of a 2F catheter.

### Cavitation threshold detection with *ex vivo* tissue

Before the *in vivo* study, an *ex vivo* study was conducted by using carotid artery plaque samples to confirm the enhanced cavitation effect for the catheter-assisted FUS approach. De-identified human carotid artery plaque samples were collected from the University of Kansas Medical Center during carotid endarterectomy interventional procedures, under an Institutional Review Board exemption. During the experiment, a 2F catheter (0.67 mm in diameter, 120602F, Edwards Lifesciences LLC, Irvine, CA, USA) was attached to the back surface of a sample, while the front surface of the sample faced the FUS transducer, and the FUS transducer was focused on the catheter wire in a water tank filled with degassed water.

Cavitation threshold was detected by aligning a passive cavitation detector (PCD) orthogonally with respect to the FUS transducer such that the focal spot of the 500 kHz FUS transducer overlapped with the focal spot of the 10 MHz center frequency PCD. Ultrasound bursts with 500 cycles were applied at the catheter–tissue interface, with PNPs varying from 0.1 to 5.0 MPa with a step size of 0.12 MPa. The acoustic scatterings of each 500-cycle ultrasound burst from the focal region of the FUS transducer were first detected by the PCD. Then, the detected signals were pre-amplified by 40 dB by using the through-mode of a pulse receiver (DPR 300, JSR Ultrasonics, Pittsford, NY, USA) before they were collected by using a digital oscilloscope (TBS2000B, Tektronix, Inc., Beaverton, OR, USA). During the data acquisition process, the high pass filter and low pass filter were kept 1 MHz and 3.5 MHz, respectively. The detection was repeated with 10 single-ultrasound bursts (*n* = 10) at each FUS pressure.

### Animal model

New Zealand White rabbits, aged 3–4 mo and weighing between 2.5 kg and 3.2 kg, both male and female, were used in this study. All animal handling procedures followed the protocol approved by the University of Kansas’ Institutional Animal Care and Use Committee (protocol number AUS 188–15, PI Dr. Xinmai Yang), adhering strictly to the National Institutes of Health guidelines.

Upon the arrival of a rabbit, the animal would be fed with a 1% cholesterol diet for 2 wk prior to a balloon injury on the left femoral artery and continued on the modified diet for 6 wk after the balloon injury until a catheter-assisted FUS treatment. Two surgeries were performed on each rabbit. The first surgery was to create the balloon injury to induce atherosclerotic plaques in the femoral artery, and the second surgery was for the catheter-assisted FUS treatment.

As a general procedure, prior to surgery, a rabbit was anesthetized with a mixture of ketamine (20 mg/kg) and xylazine (5 mg/kg). Once fully anesthetized, the hair on the lower abdomen was shaved using an electric trimmer. The rabbit was then transitioned to an isoflurane anesthesia system, where its heartbeat rate, respiratory rate and blood oxygenation were monitored. After the rabbit’s condition stabilized, it was moved to the surgical room.

During the first surgery, a balloon injury was created in the left femoral artery according to a protocol slightly modified from a published procedure [33]. A 2F balloon catheter (0.67 mm in diameter, 120602F, Edwards Lifesciences LLC) was used. The catheter was advanced into the femoral artery through an incision on the saphenous artery. The balloon was then inflated with 0.1 mL of saline solution and dragged back (~2 cm) through the femoral artery while rotating. After that, the balloon was deflated. This process was repeated three times before the catheter was removed and the saphenous artery ligated.

All rabbits would be returned to their cages and monitored for possible complications after the first surgery. All rabbits were continued on the high cholesterol diet for 6 more wk before the catheter-assisted FUS treatment.

### Treatment procedure

Six weeks after the balloon injury, the experimental system shown in Figure 1 was used to remove the atherosclerotic plaques formed in the artery. Before the treatment, the same general anesthesia procedure was followed. After the condition of the animal was stable under anesthesia, a skin incision was made to expose a portion of saphenous artery in the left leg. A 2F size catheter (the same type of catheter used to induce balloon injury to establish the animal model) was inserted into the artery and advanced to the blocked femoral artery segment. During this process, a C-arm imaging system (Fluoroscan InSight FD mini-C-Arm Imaging System, Hologic, Marlborough, MA, USA) was used to assess the blockage in the femoral artery and guide the insertion of the catheter, where X-ray contrast agent (Ioversol, 320 mg/mL) was injected manually (as needed, no more than 0.5 mL each injection) for fluoroscopy.

Ultrasound coupling gel was applied at the cone tip and the skin surface at the region of interest to provide coupling for ultrasound wave propagation into the tissue. Because the femoral artery is superficial less than 5 mm deep, the femoral artery in the depth direction would be entirely covered by the focal region of the FUS, which has a focal length of 21.42 mm. Further, by gently pressing on the skin, the position of the catheter within the vessel can be palpated. During the treatment, the transducer tip was placed at one point for 1 min and rotated gently along its axis to cover the entire catheter surface. In a similar way, the treatment was performed on multiple points along the catheter length within the artery. The spacing between treatment points was set at 3 mm, based on the focal size of the FUS transducer. The applied FUS waves were ultrasound bursts with 500 cycles, 10% duty cycle, and 100 Hz pulse repetition rate for 15 min of treatment time. After treatment, C-arm fluoroscopy was performed to detect the arterial re-canalization. In addition to the catheter-assisted FUS treatment group, an FUS-only group was also used. In the FUS-only group, no catheter insertion was made, and treatment was performed using only FUS bursts with the same acoustic parameters.

During the treatment, an ultrasound PNP of 4 MPa was applied at the tip of the conical cone. In rabbits, the femoral artery lies very close to the surface, typically at a depth of 2 to 4 mm. In addition, the skin was slightly pressed by the tip of the conical cone during the FUS treatment, further minimizing the distance between the cone tip and the artery. Consequently, the reduction in ultrasound pressure due to tissue propagation was negligible. The 4 MPa pressure was initially selected based on a previous *ex vivo* experiment results [32], where human plaques were used in FUS treatment. The study found that FUS of 4 MPa PNP did not induce cavitation and disintegrate plaques when only FUS was applied at 0.5 MHz with 10% duty cycle. The catheter-only group was an intermediate between FUS-only and catheter-assisted FUS groups, where the effect of catheter insertion into the vessel lumen was evaluated.

Due to the limitations of the animal model, we focused only on the acute effect of the FUS treatment in this study. Thus, all animals were euthanized with an intravenous overdose of pentobarbital within 30 min after the treatment, and the femoral artery in the treated area was collected for the following histologic analysis.

### Image processing and arterial re-canalization quantification

Arterial re-canalization effect after each experiment was assessed using C-arm angiography images, and re-canalization effect was segmented using the draw region-of-interest tool in the image segmentation application in MATLAB R2022b (MathWorks, Natick, MA, USA). The segmented area was binarized, and the contour area of the segmented region was calculated using the built-in MATLAB function “bwarea.” The segmentation process was performed by two engineering graduate assistants who had previous imaging process experience and were not blinded to the experiment groups. A set of original images and binarized images are shown in Figure S1.

### Statistical analysis and data visualization

One-way analysis of variance was performed by using the IBM SPSS statistical software (IBM SPSS Statistics 29.0.0.0), and re-canalization effect was visualized using the Seaborn 0.12.2 library in Python 3.11.4 (Python Software Foundation, Wilmington, DE, USA). Multiple comparisons between groups were also conducted by performing the Tukey’s test.

### Histopathological procedure and tissue imaging

#### Sample preparation for staining

Histopathological analyses were performed to confirm the atherosclerotic plaque inside the femoral artery. After euthanizing each rabbit, femoral artery sections were abscised for the control, FUS-only, and FUS-catheter group subjects. The samples were immersed in 4% formal-dehyde immediately after abscission. The paraffin-embedding procedure was performed using a Pelco BioWave Pro Laboratory Microwave System with a Pelco Steady Temp Pro Thermo Cube (Ted Pella, Inc.) (Redding, CA, USA). The dehydration process started at 40°C and involved sequentially exposing the samples to an increased concentration of alcohol for 5 min each of 50% ethanol, 70% ethanol, 95% ethanol, a 1:1 blend of 95% ethanol and isopropanol, and finally 100% isopropanol. After dehydration, samples were infiltrated with a mixture of isopropanol and paraffin (1:1) at 70°C for 5 min. They were then embedded in paraffin three times at 70°C under vacuum for 5 min, 10 min, and then 5 min. Following a 24 h period, the samples were sectioned at a thickness of 6 μm using a rotary microtome. These sections were mounted on pre-coated slides with each slide holding 8–10 sections.

Paraffin sections were subjected to de-paraffinization, hydration, staining and dehydration processes using a HistoPro 414 Linear Stainer (RUSHABH Instruments, LLC) (Ivyland, PA, USA). The de-paraffinization step involved immersing the sample in Histo-clear II (National Diagnostics, Cat. No. HS-202, Lot No. 12–23-01) (Atlanta, GA, USA) for 2 min, followed by 25 dips in Histo-clear II, repeated twice. After this, the sections were hydrated by 25 dips in a series of solutions: 100% ethanol, another 100% ethanol, 95% ethanol, 70% ethanol, and finally rinsed in running tap water for 1 min.

#### Histologic staining

The sample sections were stained with Harris Hematoxylin (EMS, Cat. No. 26754–01, Lot No. 211111–04) (Hatfield, PA, USA) for 1 min, followed by a wash in running tap water for another minute. To enhance the hematoxylin staining, the sections were dipped 25 times in Scott’s solution, which was prepared by dissolving 2.0 g of sodium bicarbonate (Sigma Life Science, Cat. No. S6014, Lot No. 127K0680) (Billerica, MA, USA) and 20.0 g of magnesium sulfate heptahydrate (Fisher Chemical, Cat. No. M63–500, Lot No. 185221) (Pittsburgh, PA, USA) in 1 L of distilled water. The sections were then rinsed in deionized water and dipped 25 times in 95% ethanol before being stained with 1% alcoholic Eosin Y (EMS, Cat. No. 26762–01, Lot No. 211111–05) (Hatfield, PA, USA) for 3 min. Finally, the tissue sections were dehydrated by dipping 25 times in 95% ethanol and then again 25 times in 95% ethanol.

#### Post-staining imaging

After this process, the samples were placed in Histo-clear II prior to mounting, and cover slips were applied using Permount mounting medium. Imaging of the sections was performed with a Nikon Eclipse LV100D-U compound bright field upright microscope. The images were captured with Q Capture Pro Version 6.0.0.412 using a Q Imaging MicroPublisher 5.0 RTV camera, with a pixel resolution of 2560 × 1920, utilizing the Plan Flour 5X/0.15NA air objective.

## Results

### Atherosclerotic model

**Figure 2.**
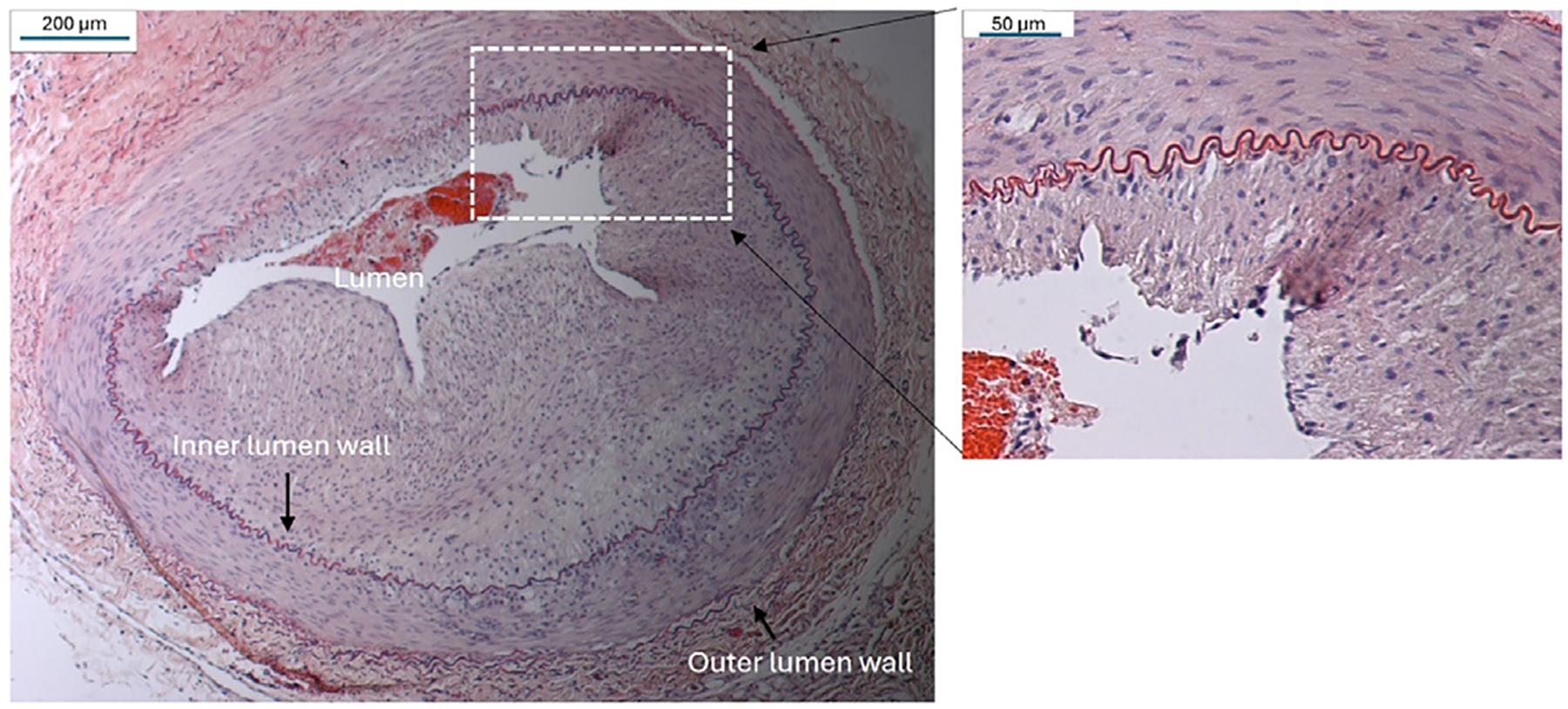
A representative histopathological photomicrograph for atherosclerotic plaque developed in a rabbit femoral artery.

Three rabbits were used to refine our technique to induce atherosclerotic plagues in the left femoral artery. Figure 2 shows a histologic section of a rabbit femoral artery, stained with hematoxylin and eosin (H&E), where a successful atherosclerotic plaque was induced 6 wk after the balloon injury. The vessel lumen was almost completely blocked by the atherosclerotic plaque components of the fibrin cap and lipid core. Please note that the atherosclerotic plaque produced by this animal model is characterized by lipid infiltrations, smooth muscle cell migration and proliferation, and recruitment of macrophages, as indicated by the previous study that established the model [33]. This image provided confirmation that the high fat diet fed to the rabbits was effective in inducing atherosclerotic plaques that constrict the blood vessel lumen, which we used as an animal model of atherosclerosis in this study.

### Cavitation threshold detection

**Figure 3.**
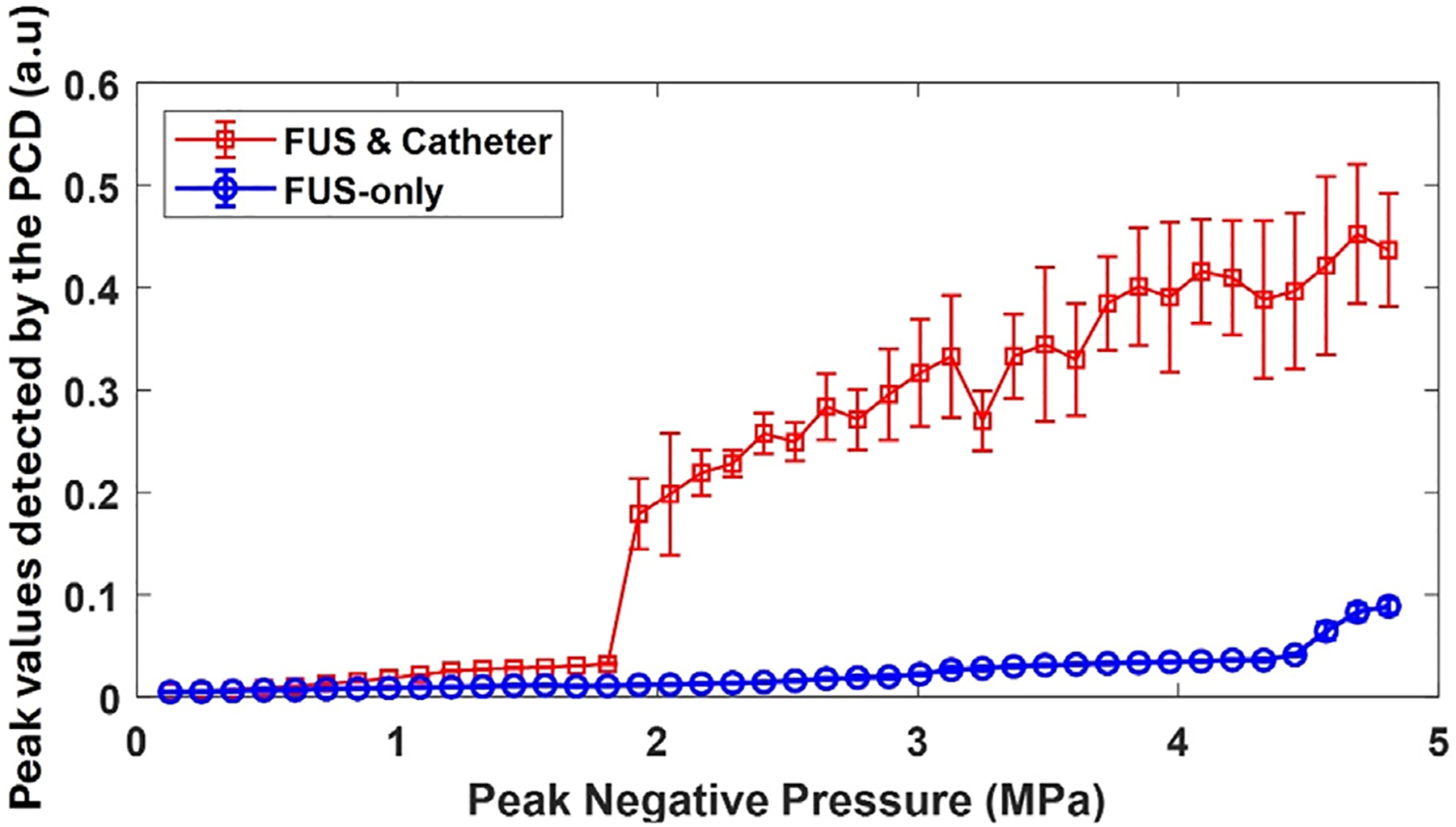
Detected peak values of signals received by the PCD under different FUS pressures in plaque samples with or without a 0.67 mm diameter catheter in the field of a pulsed FUS with 500 cycles. Error bars represent the standard deviations from 10 measurements. (a.u.) stands for arbitrary unit. *n* = 10. FUS, focused ultrasound; PCD, passive cavitation detector.

The PCD was used to measure the peak values of the scattered acoustic signals from the focal region of the FUS transducer during repetitive FUS bursts. Average and standard deviation measurements of passive cavitation signals from 10 bursts were acquired for each FUS PNP and plotted with respect to the PNP values as shown in the cavitation threshold detection curve, Figure 3, which shows a significant inflection from the linear profile in the trend of the detected peak value at 1.9 MPa FUS PNP. This phenomenon is an indication of the onset of the inertial cavitation events. The applied FUS PNP value at which this jump (more than twice the expected value on a linear profile) occurred is defined as the inertial cavitation threshold. However, the PCD results from the sample without a catheter wire showed no clear inflection for FUS PNP less than 4.4 MPa, which is an indication for the delayed onset of cavitation. These results demonstrated that the synergistic effect of FUS and a catheter could enhance the onset of cavitation and supported our selection of 4 MPa PNP for the *in vivo* experiment.

The PCD detected signal from the catheter–tissue interface decreased and fluctuated when the applied PNP was greater than the cavitation threshold pressure. A possible explanation for this phenomenon is the formation of bubbles in the pre-focal zone of the FUS transducer, which would block the FUS wave from reaching the focal region, leading to a decrease in the amplitude of scattered signals from the confocal area of the FUS transducer and the PCD. This phenomenon has been reported in previous investigations of HIFU [34]. It is also worth noting that, before the onset of cavitation, PCD showed a stronger signal strength from the sample with a catheter. This increase in signal strength is due to the scattering from the catheter itself, while the scattering from the sample without a catheter is weak before the onset of cavitation.

### Treatment on rabbit femoral artery

**Figure 4.**
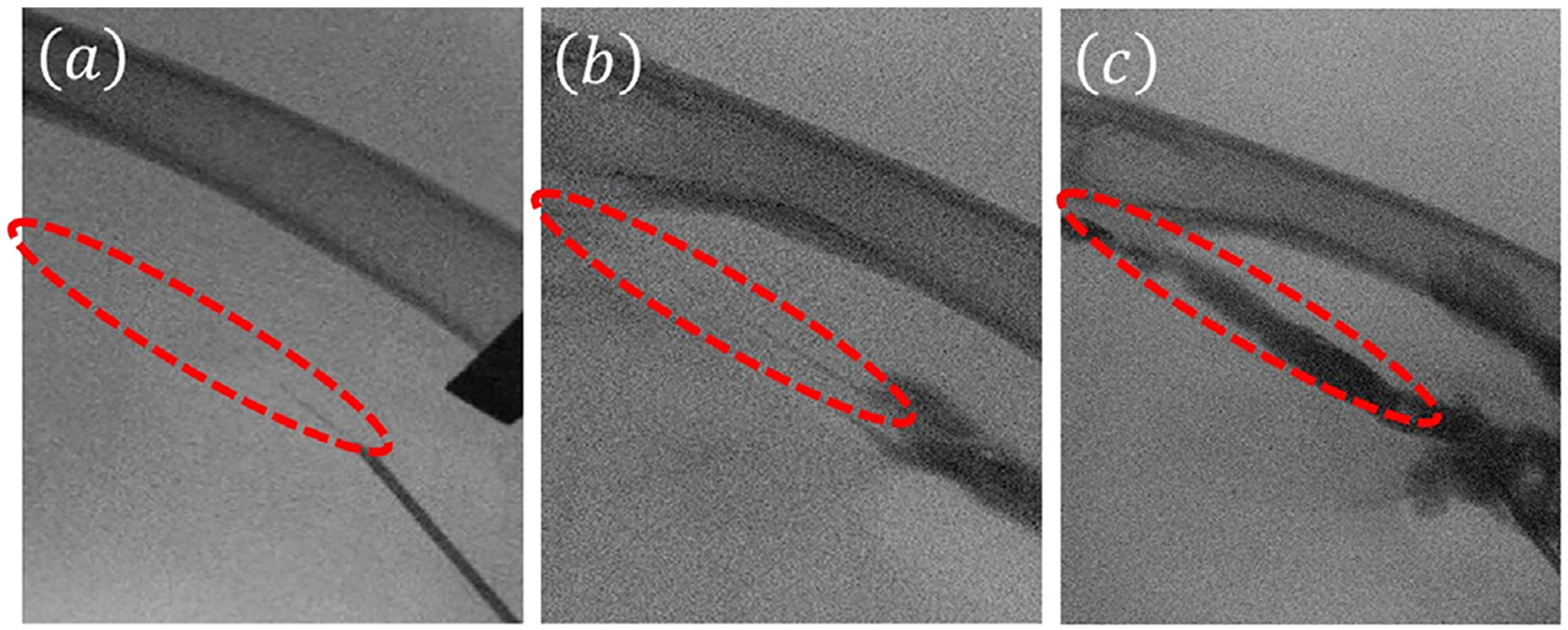
Representative C-arm angiography photographs of rabbit femoral artery atherosclerotic plaque models treated with 500 kHz FUS bursts for 15 min at 4 MPa PNP using (a) before catheter insertion, (b) after catheter insertion, and (c) after treatment using catheter-assisted FUS. Each FUS burst contained 500 cycles at 10% duty cycle with a burst repetition rate of 100 Hz. FUS, focused ultrasound; PNP, peak negative pressure.

**Figure 5.**
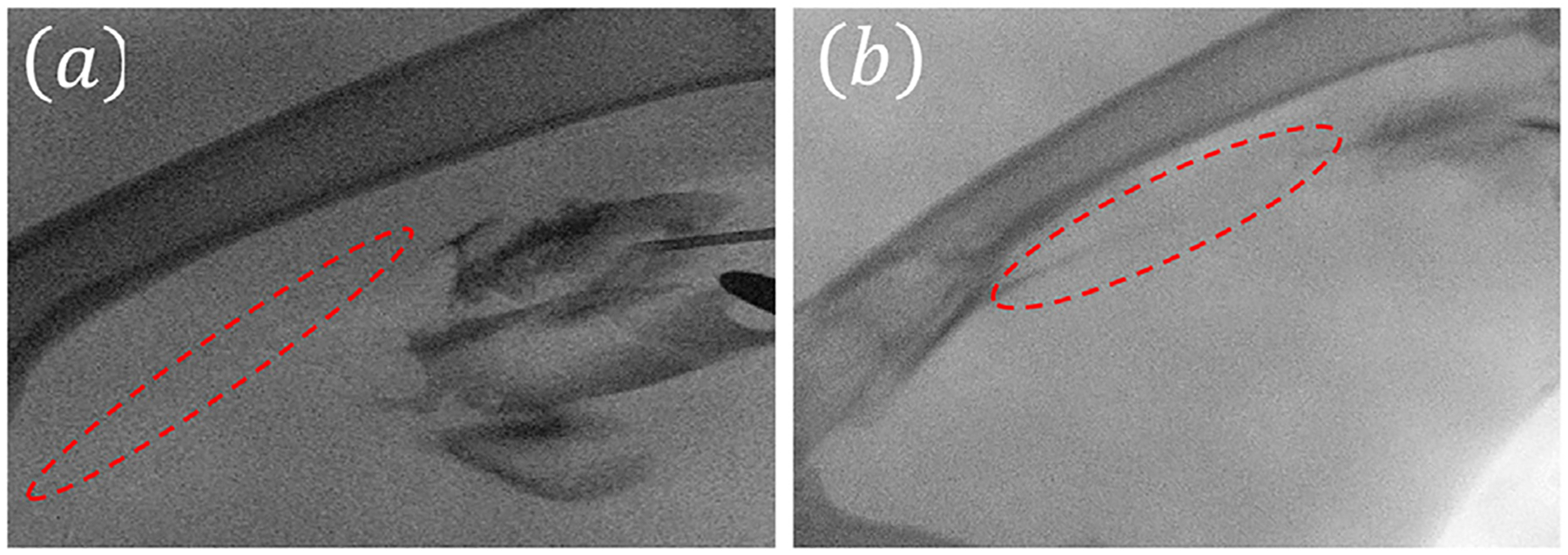
Representative C-arm angiography photographs of rabbit femoral artery atherosclerotic plaque models treated with 500 kHz FUS-only for 15 min at 4 MPa PNP using (a) before FUS therapy and (b) after FUS therapy. Each FUS burst contained 500 cycles at 10% duty cycle with a burst repetition rate of 100 Hz. FUS, focused ultrasound; PNP, peak negative pressure.

FUS therapy with and without a catheter was performed by applying 4 MPa PNP based on a process of pressure amplitude optimization as described in our previous *ex vivo* study [32]. Other ultrasound parameters were the same as the *in vitro* study. The C-arm image showed a near-complete blockage before the insertion of a catheter, as shown in Figure 4 (a). After the insertion of a catheter (without FUS treatment), the C-arm image showed an attenuated liquid flow line, as shown in Figure 4 (b). However, after the catheter-assisted FUS treatment, the C-arm image showed a wide flow channel, as shown in Figure 4 (c). In contrast, the FUS-only treatment resulted in no minimal re-canalization inside the artery after treatment as shown in Figure 5.

**Figure 6.**
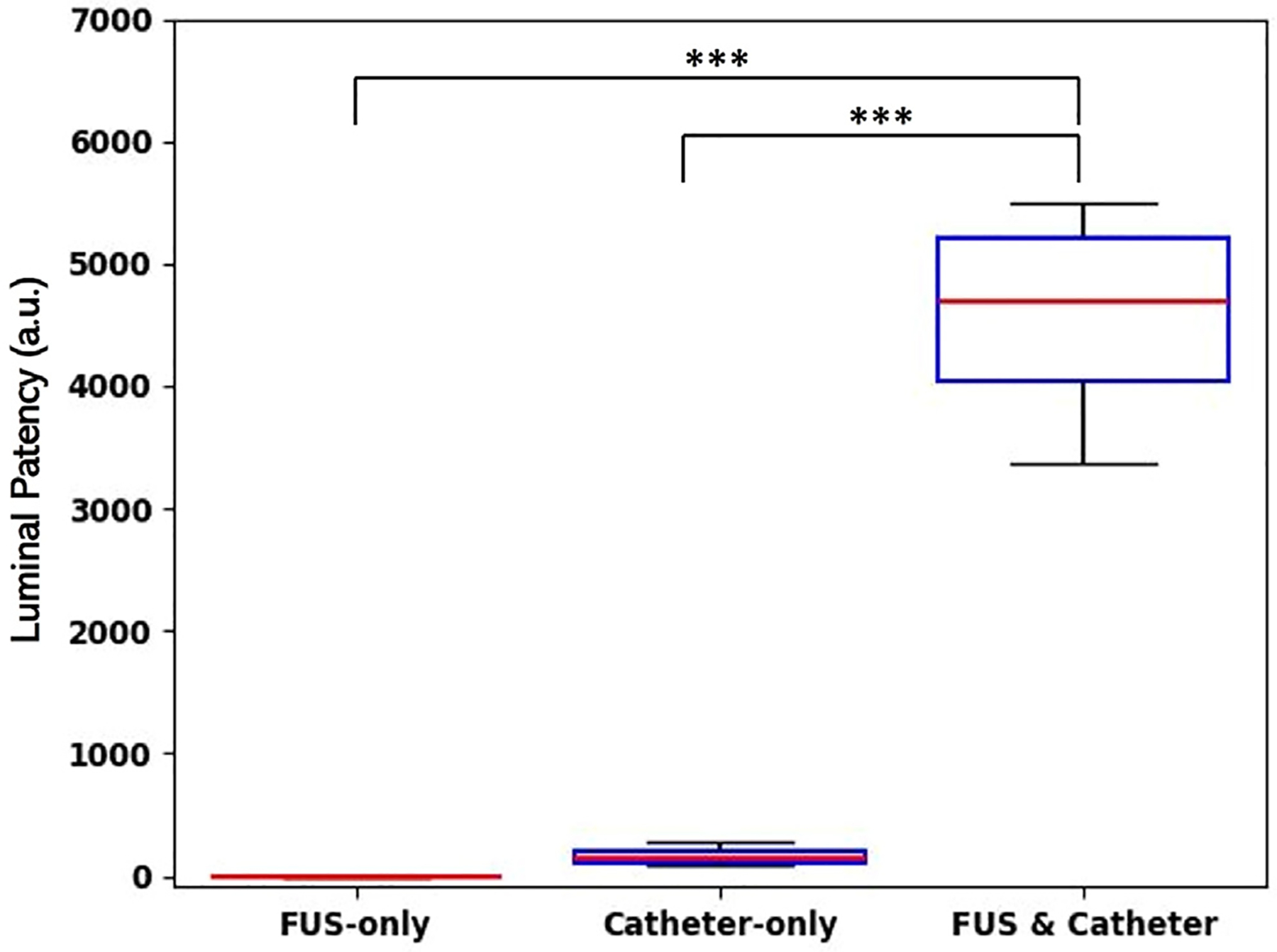
Quantification of femoral artery patency in rabbit atherosclerotic plaque models treated with 500 kHz FUS bursts for 15 min at 4 MPa PNP using FUS-only and catheter-assisted FUS. Each FUS burst contained 500 cycles at 10 % duty cycle with a burst repetition rate of 100 Hz. “***”: *p* < 0.001, *n* = 4. FUS, focused ultrasound; PNP, peak negative pressure.

Figure 6 shows the quantification of re-canalization after catheter-assisted FUS treatment using 4 MPa PNP with 500 cycles, 100 Hz burst repetition rate, and 10 % duty cycle for 15 min of therapy time. The FUS-only group showed no improvement in re-canalization with near-zero mean re-canalized area in pixels. The catheter-only group showed an area of re-canalization pixel sum of 143. Catheter-assisted FUS group showed the maximum lumen re-canalization, with a pixel sum of 1447. Statistical significance was found between groups (*p* < 0.001).

**Figure 7.**
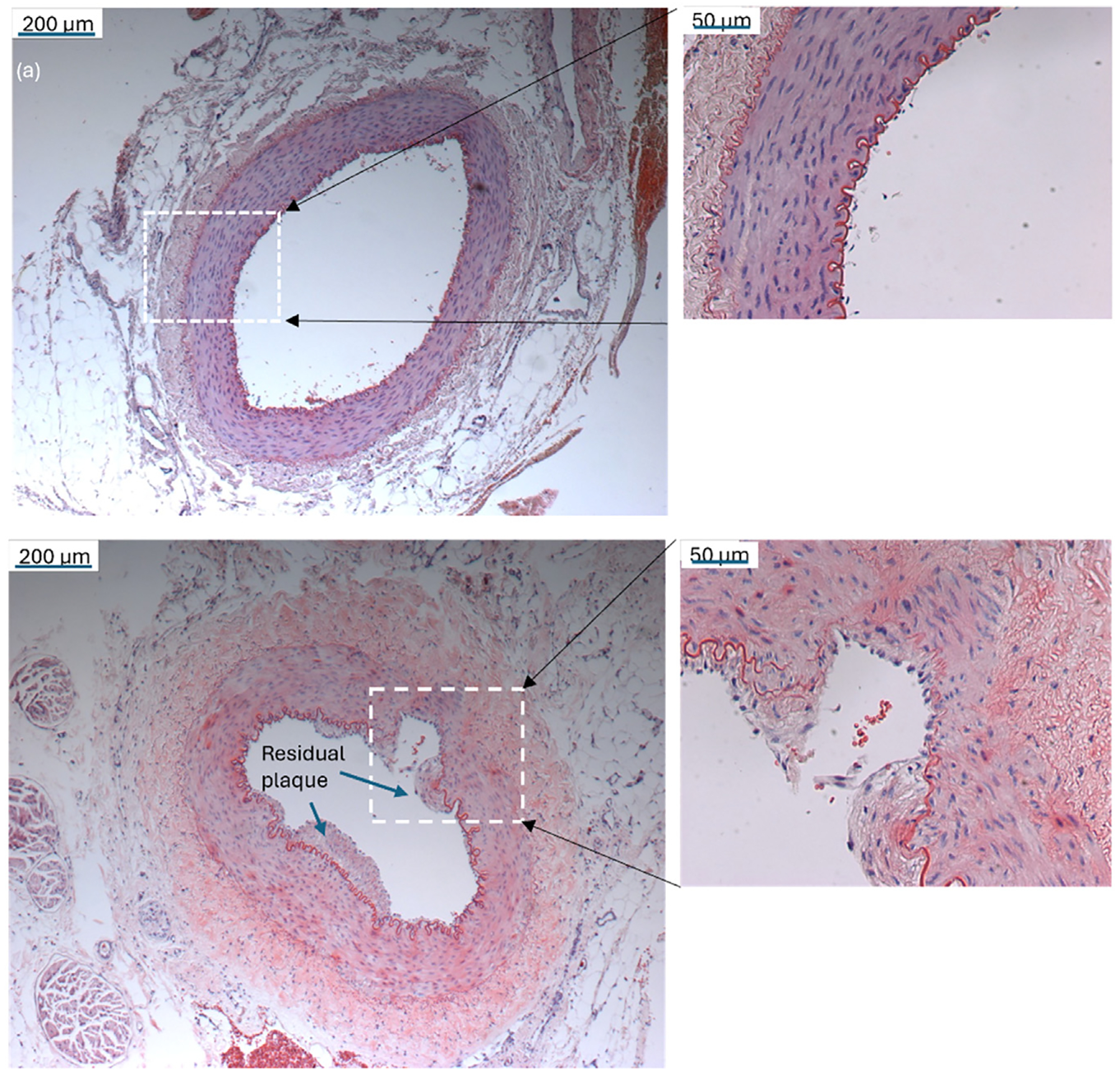
Histopathological photomicrographs after treatment using catheter-assisted FUS with 500 kHz FUS bursts for 15 min at 4 MPa PNP. Each FUS burst contained 500 cycles at 10% duty cycle with a burst repetition rate of 100 Hz. (a) A complete re-canalization of a vessel without vessel wall injury. (b) A partial re-canalization of a vessel with possible vessel injury involved in the intima and media layers. FUS, focused ultrasound; PNP, peak negative pressure.

To evaluate possible tissue damage induced by the application of catheter-assisted FUS, femoral artery cross-sections were obtained from treated rabbit subjects and stained for histopathology. Figure 7 shows examples of H&E staining results. Figure 7 (a) illustrates a complete removal of atherosclerotic plaques in the vessel without any obvious signs of acute damage to the vessel wall or adjacent tissue. Figure 7 (b) depicts a case where a few plaques remained on the vessel wall, along with potential vessel wall injuries caused by FUS-induced cavitation. The average vessel wall damage in term of area is 7.66% ± 2.21% (*n* = 5). These results indicate that while catheter-assisted FUS can effectively remove atherosclerotic plaques, further optimization is needed to enhance safety and efficiency.

## Discussion

The results from this study show that catheter-assisted FUS treatment was effective in atherosclerotic plaque disruption. In contrast, using the same level of PNP, FUS-only treatment was unable to promote re-canalization within the artery. The underlying mechanism of the catheter-assisted FUS treatment is enhanced cavitation effect as shown in Figure 3 and our previous study [32]. During catheter-assisted FUS treatment, ultrasound bursts are directed at the catheter–soft tissue interface. The catheter used in this study, the Fogarty Arterial Embolectomy Catheter 120602F, is made of polyvinyl chloride (PVC) with an acoustic-specific impedance of approximately 3.3 MRayl. In contrast, water has an acoustic-specific impedance of around 1.5 MRayl. The catheter surface can act as an acoustic scattering boundary. When superposed by incoming waves, this scattering creates a complex acoustic field influenced by factors such as acoustic pressure, wavelength, catheter size, and angle of insonation. This complex acoustic field may enhance the cavitation process. In addition, the interface between PVC and water is generally hydrophobic, leading to water repulsion and a reduced cavitation threshold at the interface.

While the catheter used in the current study is made of PVC, clinical catheters are also often made of other materials such as PEBAX or poly-urethane. These materials have acoustic properties and surface tensions that are different from PVC, which may lead to different cavitation behavior under FUS exposure. Furthermore, additional surface modifications such as surface coating or increasing surface roughness [35] may influence cavitation. As the technology continues to be optimized, these technical details should be explored in the future.

A 10% duty cycle was selected in the current study so that there is no significant thermal effect. While localized heating induced by the applied FUS is possible, we do not expect the heating effect to be significant to cause any thermal-related damage due to the use of short ultrasound bursts (1 ms) and relatively low ultrasound frequency (500 kHz). We have estimated the FUS-induced temperature rise by using an analytic formulation derived by Parker [36], which is $T(t)=\frac{\mu I}{\rho C}\left(\frac{\beta }{4D}\right)In\left(1+\frac{4Dt}{\beta }\right)$, where μ is intensity absorption coefficient of the medium, *ρ* is the density of the medium, C is the specific heat of the medium, *β* is a measure of the Gaussian distribution of the sound beam, which is assumed to have a Gaussian form of $I(r)=I_{0}e^{-r^{2}/\beta }$, and *D* is thermal diffusivity. Here, μ = 0.5 dB/cm/MHz, *ρ* = 1000 kg/m^3^, *C* = 4184 J/(Kg K), *D* = 0.11 mm^2^/s and *β* = 1.6 mm^2^. Since we treated each point for 1 min, *t* was set to 60 s. With 0.5 MHz at 4 MPa and 10% duty, the temperature increase will be around 3.8°C. This result is consistent with the fact that we did not notice thermal damage in the surrounding tissues. Please note this estimate is for the surrounding area where cavitation was not induced, whereas cavitation may cause damage when it occurs, as shown in Figure 7. To induce significant local heating, the applied acoustic pressure has to be much higher than 4 MPa that was the maximum pressure used in this study, as demonstrated in boiling histotripsy study [37].

The catheter used in this study had a diameter of 0.67 mm, and the 500 kHz FUS transducer had a focal width of 3 mm (the size of the femoral artery is 1 to 2 mm in diameter in rabbits), so the entire catheter surface was completely circumscribed by the FUS focal spot. This orientation resulted in an effective plaque depletion leading to arterial re-canalization as shown in Figure 4 (c). Previous investigations have provided evidence that it is safe in clinical settings to insert catheters of sizes smaller than 3 mm, with negligible risks to neighboring tissues in the vicinity of the blood vessel lumen [38].

In contrast to other non-invasive FUS therapies, catheter-assisted FUS uses a minimally invasive technique as the catheter is inserted within the blood vessel. However, one significant advantage of this technology is that the treatment is highly precise and localized. As the cavitation effect is narrowed to the area immediately to the catheter–tissue interface, the possibility of damaging healthy tissue is minimized. This precision in tissue removal will be imperative during atherectomy or thrombectomy events, where the blood vessel is very thin, and the occluding tissue is only a few millimeters or less in width. Another significant benefit of this technique is that cavitation nuclei are generated *in situ* at the catheter–tissue interface, and thus, no external infusion of ultrasound-contrasting agents (micro-bubbles) is required during treatment.

Since catheter-based thrombectomy and atherectomy are already well-established clinical procedures, the FUS-catheter approach does not introduce additional invasiveness. FUS can be externally applied to the catheter when removal of atherosclerotic plaque is required, resulting in only minimal deviation from current clinical practice. As such, this method holds promise as an adjunct therapy to catheter-based mechanical thrombectomy, particularly in cases where further intervention is needed to re-canalize an artery—especially in emergency scenarios where open surgery is not feasible. Another potential application is the treatment of in-stent stenosis, where the stent wire may act similarly to a catheter in facilitating FUS delivery.

This study was not without limitations. While previous studies have shown that occlusion events may not be a serious concern in cavitation-based ultrasonic treatment [15,16,21], distal occlusions are always concerns due to fragmented plaque debris that can flow further downstream of the treatment region. In the current study, the plaque debris was not systematically monitored and quantified due to the limitations of the animal model. However, further examination of the histologic results indicated distal vessel blockage possibly caused by loosened debris (Fig. S2a). In practice, since a catheter is already in place, to mitigate the possibility of distal embolism, an aspiration tube could be integrated into the catheter to aspirate out the debris.

The study indicated that potential collateral damage to the vessel wall is possible (Figure 7b) during catheter-assisted FUS. This collateral damage might be related to the cavitation activity induced in the local region. Possible nerve edema was also noticed in the nearby tissue (Fig. S2b), possibly due to the acute response to the treatment. Since cavitation activity can be controlled by applying different FUS parameters, it may be possible to minimize collateral damage by identifying the optimal FUS parameters in future studies. Particularly, optimization should also consider the effect of applied FUS frequency. Different FUS frequencies are generally related to different cavitation thresholds. In addition, the relative size between the FUS wavelength and the catheter size, as well as surface roughness, could be important factors for cavitation onset. Larger catheters may have a stronger scattering effect due to their increased surface area and affect the acoustic field differently. Further, the surface properties of the catheter should be explored. To enhance cavitation, hydrophobic surfaces are preferred, as they tend to lower the cavitation threshold. The effects of rough versus smooth surfaces may also worth investigation [35].

Another limitation of the current study is the animal model we used for testing catheter-assisted FUS. The rabbit model not only limited the size of the catheter that could be used but also limited our study to an acute study, instead of evaluating a relatively long-term effect in terms of safety. Due to the potential complications of two major survival surgeries required on the femoral artery in a long-term study, we only evaluated safety immediately after the treatment, and the evaluation was limited to possible acute vessel wall damage. A long-term study would allow us to evaluate the potential for complications caused by the debris produced during the treatment. This limitation may be addressed by using a larger animal model, such as a porcine model, in the future. With a porcine model, atherosclerotic plaques could be created in the artery though an access site on the vein, and all the vessels would be kept intact and re-accessed easily. With the current rabbit model, we have to access the femoral artery through the saphenous artery, and the saphenous needs to be ligated after the initial balloon injury in order to stop bleeding. A large animal model, such as a porcine model, would also greatly facilitate the translation of technology to human applications.

In addition, while the animal model used in the current study is well established in atherosclerosis research and has been thoroughly characterized in previous studies [33], the rabbit model used in the current study is an accelerated atherosclerosis model, which produced atherosclerotic plaques over an 8 wk period, whereas in the real world, it could take several decades for an atherosclerotic plaque to build up. While this animal model is effective for studying disease pathology, evaluating new imaging techniques and testing therapeutic agents, it does not produce heavily calcified plaques. Porcine models, on the other hand, have been developed for atherosclerosis and may produce calcified plaques [39]. However, the formation of heavily calcified plaques in porcine models requires a significant amount of time and can be very costly. Despite these challenges, using porcine models would be necessary to evaluate the proposed technique’s efficacy for heavily calcified plaques.

## Conclusion

Using an *in vivo* rabbit atherosclerotic plaque model, this study investigated the feasibility of catheter-assisted FUS treatment, leveraging enhanced cavitation effects to re-canalize a femoral artery blocked by atherosclerotic plaques. Our results demonstrated that catheter-assisted FUS can significantly deplete plaques from the vessel and re-canalize it while employing relatively low FUS PNP. In contrast, FUS-only treatment did not result in arterial re-canalization.

## Supplementary Material

1

Supplementary material associated with this article can be found in the online version at doi:10.1016/j.ultrasmedbio.2025.06.021.

## Data Availability

The data supporting the result will be made available upon reasonable request.
