# Supplementary material for: An In Vivo Study on the Feasibility of Catheter-Assisted Pulsed Focused Ultrasound Ablation of Atherosclerotic Plaques
Source: Ultrasound Med Biol. Author manuscript; Available in PMC 2026 May 4. (PMC13137374; doi:10.1016/j.ultrasmedbio.2025.06.021)
Supplement: 1 [file NIHMS2168633-supplement-1.docx]

**Supplementary Materials**


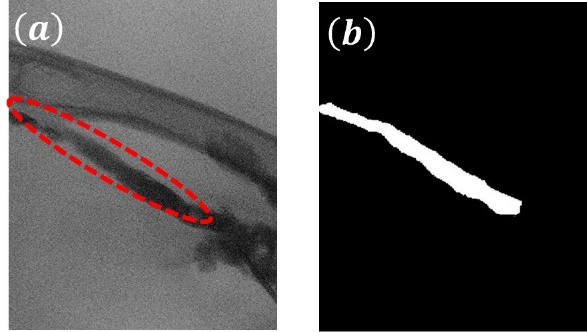


**Supplementary Figure S1**. (a) A representative C-arm angiography photograph of a treated rabbit femoral artery, (b) the corresponding binarized image for data analyses.


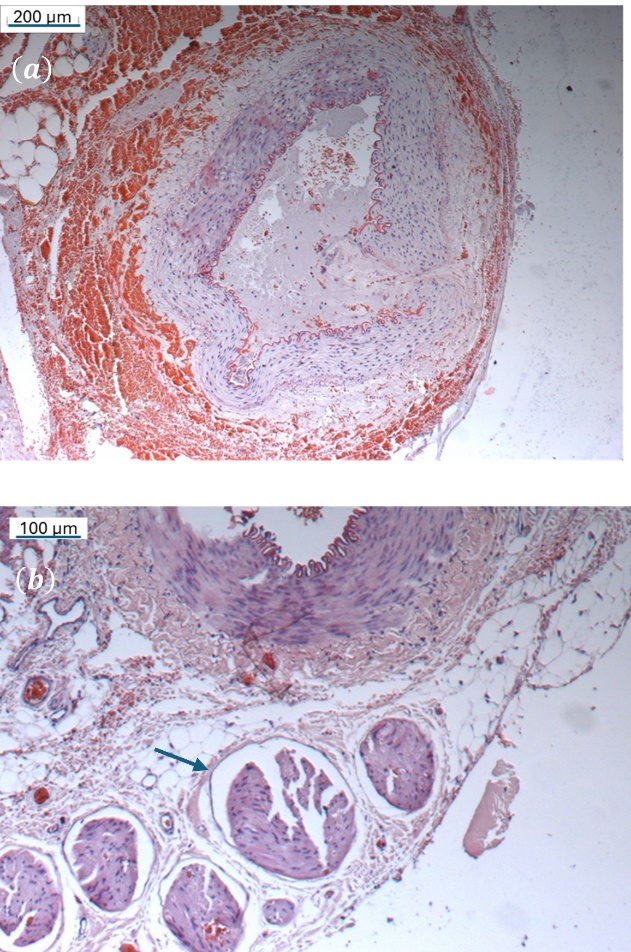


**Supplementary Figure S2**. Histological examples for further safety evaluation. (a) vessel blockage possibly caused by debris at the distal segment the treated vessel. (b) Possible nerve edema induced by the treatment.
